# TET proteins and the control of cytosine demethylation in cancer

**DOI:** 10.1186/s13073-015-0134-6

**Published:** 2015-01-29

**Authors:** Laurianne Scourzic, Enguerran Mouly, Olivier A Bernard

**Affiliations:** Institut National de la Santé et de la Recherche Médicale (INSERM), Unité 1170, équipe labellisée Ligue Contre le Cancer, 94805 Villejuif, France; Institut Gustave Roussy, 94805 Villejuif, France; University Paris 11 Sud, 91405 Orsay, France

## Abstract

The discovery that ten-eleven translocation (TET) proteins are α-ketoglutarate-dependent dioxygenases involved in the conversion of 5-methylcytosines (5-mC) to 5-hydroxymethylcytosine (5-hmC), 5-formylcytosine and 5-carboxycytosine has revealed new pathways in the cytosine methylation and demethylation process. The description of inactivating mutations in *TET2* suggests that cellular transformation is in part caused by the deregulation of this 5-mC conversion. The direct and indirect deregulation of methylation control through mutations in DNA methyltransferase and isocitrate dehydrogenase (IDH) genes, respectively, along with the importance of cytosine methylation in the control of normal and malignant cellular differentiation have provided a conceptual framework for understanding the early steps in cancer development. Here, we review recent advances in our understanding of the cytosine methylation cycle and its implication in cellular transformation, with an emphasis on TET enzymes and 5-hmC. Ongoing clinical trials targeting the activity of mutated IDH enzymes provide a proof of principle that DNA methylation is targetable, and will trigger further therapeutic applications aimed at controlling both early and late stages of cancer development.

## Introduction

DNA methylation on carbon 5 of cytosine is one of the best-studied epigenetic marks in mammals and is known to play crucial roles in cellular processes, including gene expression regulation and control of differentiation. However, variations in DNA methylation appear during normal differentiation and aging, and may contribute to tumorigenesis. The processes of DNA methylation and demethylation as well as enzymes involved in these epigenetic mechanisms have been outlined recently but still need further characterization. Concomitantly, direct and indirect deregulation of methylation control has been uncovered in human malignancies from both hematopoietic and non-hematopoietic origins.

Ten-eleven translocation (TET) enzymes are 2-oxoglutarate, oxygen- and iron-dependent dioxygenases able to catalyze the oxidation of 5-methylcytosine (5-mC) into 5-hydroxymethylcytosine (5-hmC) [1,2]. They have been identified as key players in cytosine demethylation and in the control of cellular differentiation and transformation. Acquired point mutations and deletion events targeting TET genes are frequently observed in human cancers. These mutations affect TET2 and to some extent TET3 and result in partial or total inactivation of the gene. Metabolic perturbations resulting from mutations in genes encoding isocitrate dehydrogenase (IDH), fumarate hydratase (FH) or succinate dehydrogenase (SDH) also inhibit the TET enzymes and, in turn, DNA demethylation. Deregulation of DNA methylation may also be achieved directly through mutations in genes encoding DNA methyltransferase (DNMT) [3,4]. We are now starting to understand the control of TET protein activity, their DNA targeting, and their crosstalk with other epigenetic marks. For example, several proteins that interact with TET proteins (such as O-linked β-D-N acetylglucosamine transferase (OGT)) and with methylated and oxidized cytosines have been identified, highlighting their function in the regulation of chromatin structure. Following the implementation of specific detection methods, much has recently been learned regarding the quantity and location of the oxidized cytosine forms, mainly in embryonic stem (ES) cells, and we are now on the verge of a more complete understanding of their functions.

In this review, we discuss the established and emerging roles of TET enzymes and their functions in cytosine demethylation, with an emphasis on methylcytosine and its oxidized forms in normal tissues. We assess the roles of TET enzymes in hematological cancers and solid tumors, focusing on mutations involved in TET inactivation. Finally, we discuss the potential translational applications.

## The cytosine methylation cycle

5-mC results from the transfer of a methyl group to cytosine within a CpG dinucleotide, mediated by DNMT enzymes encoded by five genes. DNMT1 is mainly responsible for the maintenance of genomic DNA methylation patterns (that is, after DNA replication), whereas DNMT2 (or tRNA cytosine-5-methyltransferase) is an RNA methyltransferase. DNMT3A and DNMT3B are mainly responsible for *de novo* DNA methylation [5]. However, all three enzymes may contribute to both maintenance and *de novo* DNA methylation [6]. The catalytically inactive DNMT3L interacts with these enzymes and the histone 3 tail to stimulate DNA methylation [7]. Furthermore, DNMT3A has recently been identified to be involved in crosstalk with epigenetic marks independently of DNMT3L [8].

**Figure 1:**
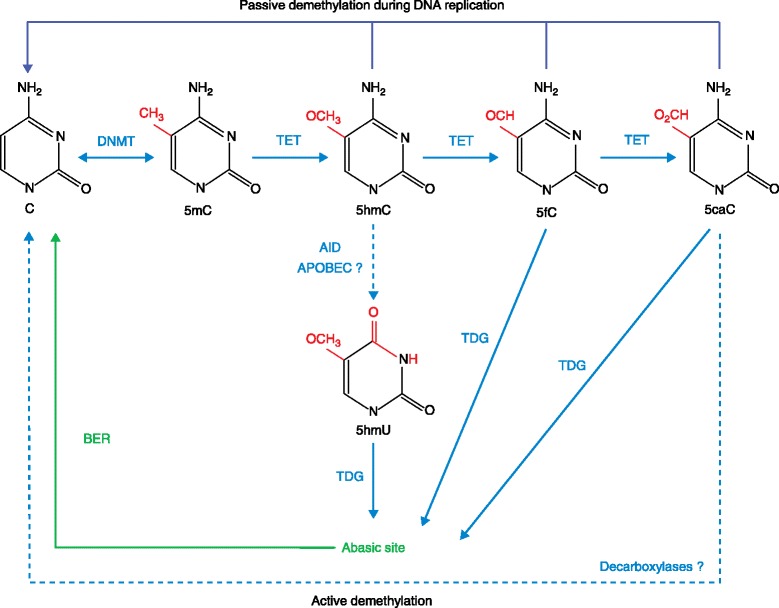
**Regulation of DNA methylation and demethylation.** DNA demethylation can occur spontaneously via the DNMT enzymes that methylated the nucleotide cytosine (5-methylcytosine, 5-mC) originally. A passive replication-dependent mechanism of DNA methylation is also possible. Several active demethylation pathways have been postulated. TET family proteins catalyze the oxidation of 5-mC into 5-hydroxymethylcytosine (5-hmC) and can further oxidize 5-hmC to 5-formylcytosine (5-fC) and 5-carboxycytosine (5-caC). 5-hmC recognition and transformation into 5-hydroxymethyluracyl (5-hmU) by activation-induced deaminase (AID) to facilitate repair by DNA glycosylase and the base-excision repair (BER) pathway is still controversial. These latter activities are also thought to process 5-fC and 5-caC into unmodified cytosine . The decarboxylases involved in this process are still to be identified. APOBEC, apolipoprotein B mRNA editing enzyme; DNMT, DNA methyltransferase; T, thymine; TDG, thymine DNA glycosylase; TET, ten-eleven translocation.

Although DNA methylation has long been recognized, and cytosine methylation by DNMT3A and DNMT3B has been shown to be reversible *in vitro* [9], the mechanism of DNA demethylation was unclear until the functional analyses of the TET family proteins [1,2]. Due to its poor recognition of 5-hmC, which results from TET activity, DNMT1 is not able to perform the methylation of the neo-synthetized DNA strand (maintenance methylation). So the methylation information is lost in dividing cells, in a so-called passive manner (Figure 1). The three enzymes of the TET family (TET1, TET2 and TET3) are able to further oxidize 5-hmC into 5-formylcytosine (5-fC) and then 5-carboxycytosine (5-caC) [10,11]. Thymidine DNA glycosylase (TDG) is then able to remove 5-fC and 5-caC, triggering base-excision repair (BER) activity and the reintroduction of unmethylated cytosine [11-13]. The existence of decarboxylases that convert 5-caC to unmethylated cytosine is hypothetical. It has been suggested that the deamination of 5-hmC into 5-hydroxymethyluracil (5-hmU) occurs via activation-induced deaminase (AID) and apolipoprotein B mRNA editing enzyme (APOBEC), followed by TDG and BER mechanisms [14]. However, this remains controversial because 5-hmU residues may also originate from TET-mediated oxidation of thymine [15]. In addition, the activity of recombinant AID decreases with the size of the cytosine C5 electron cloud and does not show any activity on 5-hmC *in vitro* [16,17]. Indeed, AID exhibits its strongest activity against unmodified cytosine. Thymine resulting from deamination of 5-mC is not easily recognized by DNA repair machinery and is considered mutagenic. These branches of the cycle need to be further investigated in a cell- and tissue-dependent context. Regardless, TET proteins as well as several other proteins (Table 1) are essential players in the demethylation of 5-mC.

**Table 1:** **Functions and expression of human and murine proteins involved in the cytosine methylation/demethylation cycle**

**Proteins**	**Functions**	**Expression levels during development and in embryonic stem cells**	**Expression levels in adult tissues**	**Expression levels in adult hematopoiesis**
TET1	Oxidization of 5-mC	High in mES cells, low in oocytes and zygotes [2,30,31]	Variable expression [56]	Low [83]
TET2	Oxidization of 5-mC	High in mES cells, low in oocytes and zygotes [2,30,31]	Widely expressed [56]	High [83]
TET3	Oxidization of 5-mC	Low in mES cells, high in oocytes and zygotes [2,30,31]	Limited expression in colon, stomach, adrenal glands and peripheral blood cells [56]	Low [83]
DNMT1	Methylation maintenance during DNA replication	High in early embryonic stages [129]	Ubiquitous [130,131]	Uniform but low in neutrophils [132]
DNMT3A	*De novo* methylation	High in early embryonic stages [129]	Ubiquitous [130,131]	Uniform but high in T lymphocytes and neutrophils [132]
DNMT3B	*De novo* methylation	High in later embryonic stages and differentiated cells [129]	Low levels except testis, thyroid and bone marrow [130,131]	Low expression except in human CD34+ cells [132]
AID	Cytidine deamination	High in immature B cells from fetal bone marrow and liver [133]	High in lymph nodes and moderated in spleen and bone marrow [134]	Mainly in activated mature B cells [134]
TDG	Glycosylation and deamination	Ubiquitous from ED 7.5 to 13.5 in mouse, high in central and peripheral nervous system, thymus, lung, liver, kidney, adrenal glands and intestine at ED 14.5 [135]	Mouse aorta [98]	Not reported
IDH1	Isocitrate decarboxylation of citric acid cycle	Not reported	Cytoplasm [92,103]	Not reported
IDH2	Isocitrate decarboxylation of citric acid cycle	Not reported	Mitochondria [92,103]	Not reported
FH	Hydration of fumarate of citric acid cycle	Not reported	Mitochondria [136]	Mature erythrocytes [136]
SDH	Oxidation of succinate of citric acid cycle	Not reported	Mitochondria [136]	Not reported

References listed in this table are based on mouse model studies, except for [56,103,83,130-133]. 5-mC, 5-methylcytosine; AID, activation-induced deaminase; DNMT, DNA methyltransferase; ED, embryonic day; FH, fumarate hydratase; IDH, isocitrate dehydrogenase; mES, murine embryonic stem; SDH, succinate dehydrogenase; TDG, thymine DNA glycosylase; TET, ten-eleven translocation.

### DNA methylation distribution and localization

CpGs represent between 1% and 4% of genomic mammalian DNA and approximately 75% of them are methylated. Most CpGs are located in repetitive DNA elements, indicating that cytosine methylation is used as a defense mechanism against transposons and other parasitic elements to maintain the function and stability of the genome [18]. CpGs are also concentrated in CpG islands, which are mainly unmethylated and are associated with DNA accessibility and gene transcription. These CpG islands are usually found close to gene promoters, and their methylation status is strongly correlated with the transcription state of the genes nearby. Methylation can also be identified within gene bodies. It preferentially occurs in a CxG context (where x can be T, A or C) in ES cells and this intragenic methylation is mainly associated with highly expressed genes [19]. Promoter and gene body methylation are being extensively investigated to elucidate specific mechanisms and factors responsible for gene expression modulation. Recently, DNMT3B was reported to be involved in the remethylation of gene body-associated genes following treatment of a colon cancer cell line with DNMT inhibitors [20].

### DNA hydroxymethylation distribution and localization

5-hmC was first identified in 1952 in bacteriophage T4 [21], and again 20 years later in the mammalian genome, in which it was found to constitute 0% to 17% of the total number of cytosine bases of brain-extracted DNA in mouse, rat and frog [22]. More recently, 5-hmC was estimated to constitute 0.6% of nucleotides in Purkinje cells, 0.2% in granule cells [1] and 0.03% in mouse ES cells [2]. However, the classical analyses of bisulfite-treated DNA do not discriminate between 5-mC and 5-hmC. Discrepancies among published studies may be due to different methodologies and analytical processes [23-26]. These studies nevertheless provide a general picture of the genome-wide distribution of modified cytosines in ES cells and other tissues.

The distribution of 5-hmC differs in several organs and tissues in mouse [27] and human [28]. The 5-hmC content also varies during development and cell differentiation [29]. For example, pluripotency correlates with high levels of 5-hmC, as observed in the inner cell mass, in multipotent adult stem cells as well as in progenitor cells. Embryonic or induced pluripotent stem cells also show a high 5-hmC level. Among differentiated cells, neuronal cells retain a high 5-hmC content [30,31]. In the blastocyst stage, erasure of DNA methylation and hydroxymethylation marks is followed by their re-establishment by TET proteins and subsequent *de novo* methylation by DNMT3A and DNMT3B. In the totipotent zygote, the paternal pronucleus shows high levels of 5-hmC [32,33] caused by genome-wide hydroxylation of 5-mC [34,35], in contrast to the low level of 5-hmC in the maternal pronucleus. This phenomenon is linked to TET3 translocation from the cytoplasm to the paternal pronucleus immediately after fertilization [36]. In addition, the maternal factor PGC7 protects 5-mC from Tet3-mediated conversion to 5-hmC by binding to maternal chromatin containing dimethylated histone H3 lysine 9 [37]. The maternal genome undergoes progressive 5-mC demethylation upon cell division. Genome-wide mapping of 5-hmC with specific antibodies or chemical labeling has enabled the design of 5-hmC distribution maps in mouse and human ES cells, as well as in neurological tissues. These studies have revealed that the 5-hmC mark is not uniformly distributed in the genome and is abundant in gene-rich euchromatin regions, particularly at promoters, exons and transcription start sites of genes expressed at low levels [38]. 5-hmC is mainly enriched in low CpG content regions and in bivalent gene promoters, characterized by both transcriptional permissive trimethylated histone H3 lysine 4 (H3K4me3) and repressive trimethylated histone H3 lysine 27 (H3K27me3) marks. Furthermore, *TET2*-mutated diffuse large B-cell lymphomas have been associated with a hypermethylation signature on gene promoters identified as bivalent in human ES cells [39]. More recently, 5-hmC was identified in intergenic regions in human ES cells. More specifically, 5-hmC was found in regions comprising *cis*-regulatory elements, such as active enhancers, with co-localization of the histone modification marks monomethylated histone H3 lysine 4 (H3K4me1) and acetylated histone H3 lysine 27 (H3K27ac), and transcription factor binding sites for pluripotency factors, such as OCT4 and NANOG, or the insulator binding protein CTCF [40,41].

### Function of oxidized cytosine forms

TET proteins participate in the regulation of gene transcription through the controlled generation of 5-hmC, 5-fC and 5-caC and their subsequent recognition by protein complexes involved in modulating chromatin structure or DNA repair [42-46].

Promoter methylation is associated with the repression of gene expression in somatic cells. It is not clear yet whether specific readers of methylated DNA, such as methyl-CpG binding protein 2 (MeCP2), or methyl-binding domain (MBD) proteins are recruited to the methylated DNA and prevent the binding of transcription factors, or if they participate directly in the establishment of compact chromatin and gene repression. MBD1, MBD2 and MBD4 preferentially bind methylated DNA, in contrast to MBD3, MBD5 and MBD6 that prefer to bind to non-methylated DNA [47], although MBD5 and MBD6 associate with heterochromatin [48]. MBD4, possessing a DNA glycosylase domain, is also involved in BER following deamination events and is able to interact with mismatch repair proteins [49,50]. Methyl-CpG binding proteins were thought to be unable to bind 5-hmC [51] until recently [43], although the ability of MBD3 to specifically bind 5-hmC [45] is still controversial [44]. The DNA damage control proteins UHRF1 and UHRF2 also have 5-mC binding ability through their SET- and RING-associated domains. Additionally, UHRF1 is able to bind hemimethylated DNA and recruit DNMT1 [52,53]; it has recently been proposed that it may also be able to bind both methylated and hydroxymethylated DNA [42]. However, these 5-mC and 5-hmC readers are rarely found to be mutated in cancer (Table 2).

**Table 2:** **Somatic mutations affecting TET genes in cancer**

**Genes**	**Mutations in solid tumors**	**Mutations in hematological malignancies**
**TET genes**		
*TET1*	Rare in endometrioid carcinoma^a^, colorectal carcinomas [105,137], lung [106,138,139] and bladder cancer [140]	Rare in AML [141] and CLL [142]
*TET2*	Rare in endometrioid carcinoma^a^, colorectal carcinomas [105,137], melanoma and lung cancer [106,138,139]	Frequent in various cancers (see Table 3)
*TET3*	Rare in endometrioid carcinoma and colorectal carcinomas [105,137]	Rare in CLL [142], PTCL [90] and T-ALL [143]
**Epigenetic regulators of TET genes**		
**Methyltransferases**		
*DNMT1*	Rare in endometrioid carcinoma^a^, colorectal carcinomas [105,137], and lung cancer [106,138,139]	Rare in AML [141]
*DNMT3A*	Rare in endometrioid carcinoma^a^, colorectal carcinomas [105,137], lung cancer [106,138,139] and 2% in non-small cell lung cancer [144]	AML [128], MDS [145] and T-cell lymphomas [90,91]
**Deaminase and glycosylase**		
*AID*	Rare in glioblastoma and medulloblastoma [146], endometrioid carcinoma^a^, colon cancer [105,137] and lung cancer [106,138,139]	T-ALL [147]
*TDG*	Rare in endometrioid carcinoma^a^, rare in glioblastoma^a^, colon cancer [104,136], lung [106,138,139] and thyroid cancer [148]	Not reported
**Histone crosstalk regulators**		
*OGT*	Rare in endometrioid carcinoma^a^, colorectal carcinomas [105,137], lung cancer [106,138,139] and breast cancer [115,149]	DLBCL [150] and CLL [142]
*IDAX*	Rare in breast cancer^a^, glioblastoma^a^, endometrioid carcinoma^a^, kidney^a^, colon cancer [105,137], lung cancer [106,138,139] and 1.2% in mouth and pharynx carcinoma [117]	Not reported
**5-mC and 5-hmC readers**		
*MBD1*	Rare in endometrioid carcinoma^a^, colorectal carcinomas [105,137], lung cancer [106,138,139], breast cancer [149] and melanoma [109]	Rare in ALL [151]
*MBD4*	Rare in endometrioid carcinoma^a^, colorectal carcinomas [105,137], lung cancer [106,138,139], breast cancer [115] and melanoma [109]	Rare in AML [141]
*UHRF1*	Rare in endometrioid carcinoma^a^, colorectal carcinomas [105,137] and lung cancer [106,138,139]	Rare in B-ALL [152]
**Other genes affecting TET functions**		
**Metabolic enzymes**		
*IDH1*	Rare in paragangliomas [153], frequent in chondrosarcomas [154], thyroid [155,156], prostate [157] and central nervous system cancers [102,158,159]	Frequent in AML [88], MDS [160], DLBCL [161] and B-ALL [162]
*IDH2*	Rare in endometrioid carcinoma^a^ and colorectal carcinomas [105,137], frequent in chondrosarcomas [154] and central nervous system cancers [102,158,159]	Frequent in AML [88], MDS [160] and AITL [89]
*FH*	Renal cell carcinoma [163] and paragangliomas [104]	Not reported
*SDH*	Renal cell carcinoma [164] and paragangliomas [104]	Not reported

^a^COSMIC database. Some mutations listed in this table have not been confirmed as somatic mutations. 5-mC, 5-methylcytosine; 5-hmC, 5-hydroxymethylcytosine; AID, activation-induced deaminase; AML, acute myeloid leukemia; AITL, angioimmunoblastic T-cell lymphoma; B-ALL, B-cell acute lymphoblastic leukemia; CLL, chronic lymphocytic leukemia; DLBCL, diffuse large B-cell lymphoma; DNMT, DNA methyltransferase; FH, fumarate hydratase; IDAX, Inhibition of the Dvl and Axin complex; IDH, isocitrate dehydrogenase; MBD, methyl-binding domain; MDS, myelodysplastic syndrome; OGT, O-linked β-D-N acetylglucosamine transferase; PTCL, peripheral T-cell lymphoma; SDH, succinate dehydrogenase; T-ALL, T-cell acute lymphoblastic leukemia; TDG, thymine DNA glycosylase; TET, ten-eleven translocation; UHRF, ubiquitin-like with PHD and ring finger domains.

In ES cells, the distributions of 5-fC and 5-caC resemble those of 5-hmC, with a preference for enhancers, and bivalent and silent promoters. Analyses of proteins interacting with cytosine-oxidized forms have identified glycosylase and DNA repair proteins interacting with 5-fC at a higher level compared with other cytosine forms, suggesting that 5-fC may trigger repair-associated removal [44].

## TET proteins

**Figure 2:**
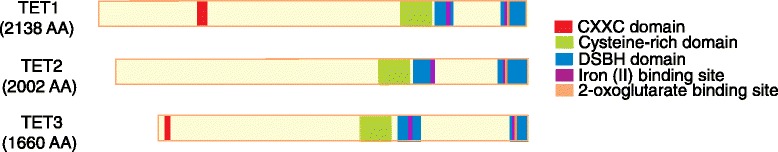
**Primary structure and function of human TET proteins.** All TET proteins present a double-stranded β helix (DSBH), a cysteine-rich domain, and one 2-oxoglutarate and three iron (II) binding sites in the carboxyl terminus, which constitute their dioxygenase catalytic domain. An amino-terminal CXXC zinc finger domain is only identified in TET1 and TET3, allowing these enzymes to bind DNA directly to CpG. Recently, the CXXC4 gene (also named inhibition of the Dvl and Axin complex, *IDAX*), located upstream of TET2 on chromosome 4, has been reported to tether TET2 to DNA through a physical interaction [65]. AA, amino acid; TET, ten-eleven translocation.

TET1 was first identified as a rare fusion partner of the mixed lineage leukemia gene, resulting from the chromosomal translocation t(10;11)(q22;23) in acute leukemia [2,54-57]. The difference between TET proteins relies on their structure (Figure 2) but also on their distinct expression patterns: TET2 is more highly expressed in the hematopoietic system than TET3 and TET1. It is currently thought that the common and main function of TET proteins is to establish or maintain protective boundaries to prevent unwanted methylation of non-methylated regions [58]. Each TET protein may also have specific functions: for example, TET1 oxidizes 5-mC to 5-hmC, and TET2 and TET3 stimulate the removal of 5-hmC [59]. In ES cells, TET2 may preferentially act on gene bodies, and TET1 at promoters [60]. The role of TET-mediated cytosine oxidation at distal enhancers is currently being thoroughly investigated. Super enhancers (enhancer clusters) that produce enhancer-transcribed RNAs in mouse ES cells have recently been associated with H3K27ac, TET1 and a decrease in DNA methylation level at pluripotency-dedicated loci [61]. Also, a specific role for TET2 in the control of enhancer activity has been suggested in the context of murine ES cell differentiation [62]. This mechanism remains to be investigated in the context of cancer, and more specifically in hematological disorders.

### Interaction with other proteins

The stability and activity of TET proteins are regulated in several ways. Vitamin C has been reported as a cofactor that enhances the activity of TET enzymes [63,64]. The Dvl-binding protein inhibition of the Dvl and Axin complex (IDAX) can recruit TET2 to unmethylated DNA via the CXXC domain, and at the same time is able to induce its proteolytic degradation by caspase activation [65]. Other proteins interact with TET proteins, such as early B-cell factor 1 [66], or modulate their subcellular localization, such as AID [67], but it is not yet clear whether they affect TET stability and function. This is also the case for OGT, which can associate with TET proteins [68-70] but appears to differently affect the three proteins. For instance, OGT has been described to trigger the export of TET3 from the nucleus and thus impair its activity [71]. A better understanding of multiple TET functions will arise from the identification of TET partners in normal and cancerous cellular contexts.

### Crosstalk with other epigenetic mechanisms

In addition to transcriptional regulation through the readers of 5-hmC, 5-fC and 5-caC, another level of transcriptional regulation mediated by TETs comes from the interplay between DNA and histone modifiers. TET1 has been shown to interact with histone deacetylases through the transcriptional corepressor SIN3 transcription regulator family member A, thereby promoting transcriptional repression [72]. TET proteins can recruit OGT enzymes to chromatin, which catalyzes the addition of O-linked β-D-N acetylglucosamine to serine and threonine within histones and other proteins. TET proteins also interact indirectly with the complex proteins associated with SET1 (COMPASS) complex, which is responsible for mono-, di- and trimethylation of histone 3 lysine 4 and is associated with active transcription. This interaction occurs through the OGT-mediated glycosylation of the COMPASS subunit host cell factor 1. The COMPASS complex of proteins is involved in the regulation of master genes, such as *HOX*, during development, balanced by the action of the polycomb repressive complex (PRC), which catalyzes the repressive mark H3K27me3. In addition, TET1 shares target genes with PRC2 in ES cells [73]. In conclusion, TET proteins also serve as platforms for other epigenetic activities [74].

### Other TET functions

The TET family is conserved during evolution. *Drosophila*, for example, has one homologous gene, whose function remains undetermined because of the particular DNA methylation pattern of flies [75]. Additional TET functions might be uncovered in the future, and a recent report indicates that mammalian TET proteins may catalyze the formation of 5-hydroxymethylcytidine *in vitro*, suggesting a role in RNA modification [76]. Recently, TET triple knockout mouse ES cells were generated using the CRISPR/Cas9 system, suggesting a novel function of these proteins in telomere length regulation [77]. Indeed, triple knockout ES cells have an increased telomere length associated with a higher frequency of telomere-sister chromatid exchange. Although TET proteins seem to be involved in telomere shortening, their precise roles need to be further investigated in the context of both normal and cancerous cells.

## TET and cancer

Here, we discuss the role of TET proteins in cancer focusing on TET2 mutations and activity impairment, first in hematopoietic malignances and then in solid tumors.

### TET in hematopoietic malignancies

#### TET mutations

Inactivation of *TET2* by genomic deletions or mutations has been reported in a wide range of adult hematological malignancies, including acute myeloid leukemia (AML), myelodysplastic syndrome (MDS) and myeloproliferative neoplasms (MPN) [78-80], as well as in lymphoid malignancies [39,81] (Table 3). In myeloid malignancies, *TET2* mutations are associated with a decrease in 5-hmC levels and an increase in 5-mC levels with respect to TET2-wild-type samples [82-84]. Many *TET2* acquired missense mutations have been described. Mutations that target the evolutionarily conserved catalytic domain of the protein are predicted to impair its function. Other missense mutations, occurring, for example, in the amino-terminal part of the protein, may also affect its function in an as yet uncharacterized manner. *TET2* mutations are observed on only one of the two gene copies, indicating that partial inactivation of the protein may contribute to cellular transformation [78]. There are marked differences between the three *TET* genes in terms of their expression levels. *TET2*, for example, has a higher expression level in hematological cells than *TET1* or *TET3. TET3* expression levels are higher than *TET1* levels in hematopoietic progenitor cells. Mutations in *TET3* have also been described but are much less frequent, probably because of its lower expression in hematopoiesis. Regarding *TET1*, most of the currently described mutations are missense mutations, whose functional consequences have not been established.

**Table 3:** **Prevalence of** ***TET1*** **,** ***TET2*** **and** ***TET3*** **mutations in hematological malignancies and solid tumors**

**Cancer**	***TET1*** **mutation prevalence (%)**	***TET2*** **mutation prevalence (%)**	***TET3*** **mutation prevalence (%)**
**Myeloid malignancies**			
**MDS**	Not reported	6-26 [78,79,165-169]	Not reported
**MDS/MPN**			
CMML (adult)	Not reported	20-58 [78,169-174]	Not reported
**MPD**			
PV	Not reported	6-16 [78,169,170]	Not reported
ET	Not reported	4-5 [78,169,170]	Not reported
MF	Not reported	2-17 [78,169,170]	Not reported
**CML**	Not reported	2-4 [175,176]	Not reported
**AML**			
*De novo* (adult)	Rare [141]	12-27 [169,170,177-181]	Not reported
*De novo* (pediatric)	Not reported	2-4 [182,183]	Not reported
Secondary AML	Not reported	17-32 [80,85,184,185]	Not reported
**Mastocytosis**	Not reported	20-29 [87,186]	Not reported
**BPDCN**	Not reported	25-54 [187-189]	Not reported
**Lymphoid malignancies**			
**B-cell lymphoma**			
DLBCL	Not reported	6-12 [39,81]	Not reported
MCL	Not reported	0-4 [81,190]	Not reported
Follicular lymphoma	Not reported	2 [81]	Not reported
**CLL**	Rare [142]	Not reported	Rare [142]
**T cell lymphoma**			
AITL	Not reported	33-83 [81,86,91,191,192]	Not reported
PTCL and PTCL, NOS	Not reported	20-49 [81,86,91,191,192]	Rare [90]
**T-ALL**	Not reported	Not reported	Rare [143]
**Solid tumors from**			
**Endometrium**	9*	7*	4*
**Breast**	Rare [106]	Rare [115]	Rare*
**Central nervous system**	Rare [193]	Rare*	Rare*
**Kidney**	Rare*	Rare [113]	Rare*
**Large intestine**	7 [105,137]	4 [105,137]	5 [105,137]
**Liver**	Rare [194]	Rare*	Rare [195]
**Lung**	5 [106,114,138]	2 [115,138,139]	Rare [138,139]
**Ovary**	Rare*	Rare*	Rare [112]
**Pancreas**	Rare [196,197]	Rare*	Rare*
**Prostate**	Rare [106,198]	Rare [198,199]	Rare [199]
**Skin**	Rare [109]	1 [109]	Rare [116]
**Stomach**	4 [200,201]	Not reported	Rare*
**Urinary tract**	4 [140]	4*	Rare*

AITL, angioimmunoblastic T cell lymphoma; AML, acute myeloid leukemia; BPDCN, blastic plasmacytoid dendritic cell neoplasm; CLL, chronic lymphocytic leukemia; CML, chronic myeloid leukemia; CMML, chronic myelomonocytic leukemia; DLBCL, diffuse large B cell lymphoma; ET, essential thrombocytosis; FL, follicular lymphoma; MCL, mantle cell lymphoma; MDS, myelodysplastic syndrome; MF, myelofribrosis; MDS/MPN, myelodysplastic syndrome/myeloproliferative neoplasm; MPD, myeloproliferative disorder; PV, polycythemia vera; PTCL, peripheral T cell lymphoma; PTCL,NOS, peripheral T cell lymphoma not otherwise specified; T-ALL, T-cell acute lymphoblastic leukemia; TET, Ten eleven translocation. *COSMIC database. Some mutations listed in this table have not been confirmed as somatic mutations.

#### Associations with other mutations

Mouse and human studies have shown that the loss of *TET2* endows cells with a growth advantage over wild-type cells, but does not lead to full transformation. Although this is not always the case, *TET2* mutation frequently occurs before the *JAK2*^*V617F*^ mutation in the development of MPN [78,85], suggesting that *TET2* mutation may occur very early in cancer development. TET2 mutations also occur in early progenitors in MDS. Acquired *TET2* mutations are also observed in lymphoma, both B- and T-cell types, and particularly in angioimmunoblastic T-cell lymphoma (AITL). In both T- and B-cell lymphomas, *TET2* mutations have been identified in multipotent progenitors [86] that are able to participate in both myeloid and lymphoid differentiation. Together, these observations indicate that *TET2* loss predisposes but does not trigger cellular transformation. The tumor phenotype depends on cooperating mutations, such as *JAK2* or *KIT* mutations for MPN [87].

In AML, *TET2* mutations occur with other major mutations, particularly internal tandem duplication of *FLT3*, as well as mutations in *RAS*, *NPM1* and *DNMT3A*. Mutations in *TET2*, *IDH1* and *IDH2* are, however, mutually exclusive [88]. The situation is markedly different in AITL. Here, *TET2* mutations are closely associated with *DNMT3A* mutations [86] and, even more intriguing, do occur together with *IDH2* mutations [89-91].

#### TET and IDH mutations

IDH mutant proteins can inhibit TET2 activity. The *IDH* genes encode enzymes of the citric acid cycle that convert isocitrate into α-ketoglutarate (αKG) in a nicotinamide adenine dinucleotide phosphate-dependent manner. A variety of human cancers, including AML [92-94], show recurrent missense mutations in *IDH1* and *IDH2* that endow the mutant protein with the ability to synthesize 2-hydroxyglutarate (2HG) from αKG (Table 2). 2HG is a competitive inhibitor of αKG and may inhibit all αKG-dependent dioxygenases, including EGLN prolyl hydroxylases, Jumanji C histone demethylases and TET proteins. In AML, *TET2* and *IDH* mutations are mutually exclusive, suggesting that they target the same pathway [84]. Consistent with this, *TET2*- and *IDH*-mutated primary AML samples show comparable DNA methylation profiles [84,95].

#### Other examples of TET2 activity targeting in myeloid malignancies

A recent report indicates that mutations in the *WT1* gene are exclusive from *TET2*, *IDH1* and *IDH2* mutations and impair TET2 activity in human AML. The *WT1* gene encodes a zinc finger transcription factor and is mutated in approximately 8% of patients. Similar to patients with mutations in *IDH1*, *IDH2* and *TET2*, samples from patients with *WT1-*mutated primary AML show decreased 5-hmC levels and changes in 5-hmC localization. This study indicates the involvement of WT1 in the regulation of hydroxymethylation and provides an example of TET2 function impairment without *TET2* mutations [96].

Mouse models have shown that microRNAs (miRNAs) miR26a and miR29a are able to regulate TET expression by targeting their 3’ untranslated regions (UTRs) [97,98]. Other miRNAs, such as miR125b, miR29b, miR29c, miR101 and miR7, have also been implicated in TET regulation using a 3’ UTR human and mouse reporter screen [99]. Recently, miR22 has been shown to be responsible for the downregulation of all three TET genes [100]. Indeed, conditional expression of miR22 in a transgenic mouse model led to reduced levels of 5-hmC, amplification of the hematopoietic stem/progenitor compartment, and development of hematopoietic malignancies. miR22 is highly expressed in more than half of adult MDS and AML samples, providing another example that TET2 activity can be knocked down in the absence of a somatic mutation.

### 5-hmC and TET in solid tumors

#### Deregulation of cytosine hydroxymethylation by TET activity

Abnormal patterns of cytosine methylation have been observed in some solid tumors, including melanoma. The melanoma epigenome widely lacks 5-hmC, in association with tumor progression and downregulation of the *TET* family genes [101]. However, somatic *TET* mutations are exceedingly rare in this cancer, suggesting that another mechanism is affecting TET activity. Considering that TET enzymes are dependent on αKG, alteration in genes participating in its production may contribute to the inhibition of TET activity. Accordingly, *IDH1* or *IDH2* mutations are described in 10% of melanomas. These data support a role for deregulation of DNA methylation control during tumor progression rather than during the initial phases.

*IDH* mutations were first observed in human gliomas [102]. The *IDH*-mutated samples exhibited a hypermethylation phenotype, due to the inactivation of TET proteins by 2HG [103]. In paragangliomas, inactivating mutations in the *SDHx* and *FH* genes, encoding citric acid cycle enzymes (Table 2), result in the accumulation of succinate or fumarate, respectively, and competitive inhibition of αKG-dependent dioxygenases, similar to 2HG [104]. *SDH* mutations induce a hypermethylation phenotype compared to tumors with wild-type *SDH*, and are associated with transcriptional silencing. This argues for a driver role for demethylation deregulation in the development of these tumors.

#### TET mutations

*TET* mutations are rare in solid tumors [105-117]. In many instances, acquired mutations are missense mutations whose functional consequences on TET protein activity are uncertain. A survey of *TET2* mutations in the COSMIC database showed more deleterious mutations in hematological malignancies than in solid tumors (29.8% versus 7.3% for frameshift mutations and 28.1% versus 10.3% for nonsense mutations). Conversely, there are fewer potentially benign mutations in hematological malignancies than in solid tumors (0.25% versus 17.6% in solid tumors for coding-silent mutations and 26.5% versus 63.1% for missense mutations). The dominant expression of *TET2* (with respect to *TET1* and *TET3*) in hematopoiesis results in a strong effect of *TET2* deficiency on 5-hmC levels. Aside from the potential specific functions of *TET2*, because expression of the three *TET* genes is equivalent in other tissues, the consequences of *TET2* deficiency on global cytosine (hydroxy)methylation is expected to be less important than in hematopoietic tissues. *IDH*, *SDH* and *FH* mutations, which result in the inhibition of virtually all αKG-dependent dioxygenases, including all three TET proteins, would therefore more strongly impact DNA methylation control than a single *TET* gene mutation.

## Implications for disease

Studies of TET2 deficiencies in tumor development have revealed the importance of DNA methylation in cellular processes as well as in the progressive development of adult type hematological malignancies.

In terms of potential clinical applicability, it appears difficult to specifically and directly target these TET dioxygenases for cancer treatment because they are inactivated in cancer. Indeed, recent efforts have focused on indirect correction of TET function and 5-hmC deregulation in cancer.

TET inactivation induces a methylation imbalance, including hypermethylation of tumor suppressor genes in malignant clones. These genes may be targeted by hypomethylating agents already used in clinical studies, such as 5-azacitidine and decitabine [118,119]. The global hypomethylation effect of these drugs, which remains nonspecific, seems to be accompanied by local hypermethylation, whose long-term consequences are unknown [20]. The molecular mechanisms of action of these drugs need to be further investigated, and extensive clinical trials are needed to prove their efficacy and to identify biomarkers of clinical responses.

In *IDH1-* or *IDH2-*mutated cancers, the oncometabolite 2HG acts as a biomarker of compromised enzyme activity [120]. This led to the development of IDH2 inhibitors, now tested in clinical trials [121]. Similarly, FH and SDH inhibitors could be developed to prevent the overall effect of metabolic TET inactivation in cancer. The activities of TET as well as DNMT enzymes are regulated, in part, by the concentrations of their required cofactors. Thus, the metabolic state of the cell is an antitumor target, by preventing the activity of the mutated protein but also by manipulating agonist or antagonist functions. In addition to the detection of *TET2* mutations that pre-date full-blown malignancies, recent studies have highlighted preleukemic phases in AML that are associated with mutations in other genes affecting DNA methylation, such as *DNMT3A*, *IDH1* and *IDH2* [122], and in genes involved in chromatin structure, such as *SMC1A* (structural maintenance of chromosome 1A) [123]. These observations suggest that manipulating the control of chromatin structure may be efficient for the treatment of both early and late phases of disease.

## Conclusions and future directions

**Figure 3:**
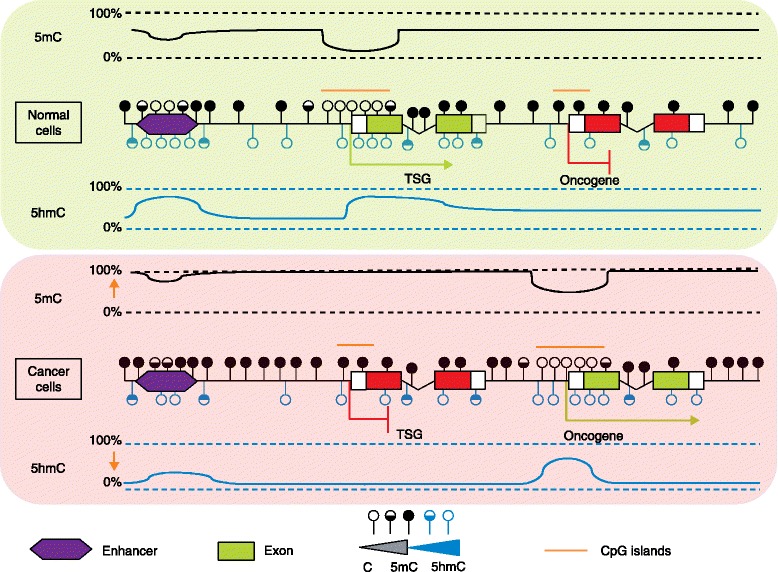
**Schematic of methylation and hydroxymethylation patterns in normal and cancer cells.** In normal cells, unmethylated cytosines are located in CpG islands and promoters of actively transcribed genes, whereas promoters of repressed genes are mainly composed of 5-methylcytosine (5-mC). 5-hydroxymethylcytosines (5-hmCs) are frequent in *cis*-regulatory elements such as enhancers, in low CpG content regions, and within gene bodies of transcribed genes. A global hypermethylation phenotype, with respect to normal tissue, is usually associated with tumoral transformation, including repressed tumor suppressor gene promoters. Hypomethylation can, however, be observed locally, in oncogene promoters, for example. Cancer cells present a global decrease of 5-hmC and local redistribution of this mark towards some oncogene promoters. C, cytosine; TSG, tumor suppressor gene.

DNA methylation patterns are markedly abnormal in malignant cells in comparison with normal tissues. Abnormal methylation has been postulated to inactivate tumor suppressor genes through cytosine methylation and to activate oncogenes through cytosine hydroxymethylation and demethylation (Figure 3). An unexpected number of oxidized cytosine forms have been uncovered, whose specific functions need to be investigated. Specific techniques allowing their thorough investigation at the nucleotide level are under development and will enable us to investigate the specific functions of these cytosines in normal cells. This is a requirement for understanding their roles in cellular transformation, because mutations detected in cancer can inactivate or impair DNA methylation (for example, *DNMT3A* mutations) or DNA demethylation (for example, *TET2* or *IDH* mutations).

Although cytosine methylation is associated with gene repression, the exact mechanisms leading from methylation to gene repression are still elusive, as are the crosstalk with other epigenetic modifications, the factors involved in chromatin modification and the regulation of their activities. DNA methylation and demethylation result from the regulation of different enzymatic activities, which compete with each other for DNA access.

This process is complex enough to appear stochastic, with a slow drift in methylation profiles that is tissue specific as well as age related. This drift leads to cellular heterogeneity and, with respect to methylation and gene repression, allows for cell selection. It is likely that TET2 deficiency increases cellular heterogeneity and facilitates the selection of fitter cells. We now require a complete understanding of the protein complexes involved in cytosine methylation and demethylation, including the exact role of each of the mammalian TET proteins and their regulatory signals, in order to target these processes.

Work with human samples and murine models has shown that *TET2* deficiency does not trigger full-blown malignancies but predisposes to the development of tumors such as MPN, MDS and lymphoma. The different *Tet2* knockout mouse models exhibit similar phenotypes but also present subtle differences that might be due to the loss of different regions of the gene. So far, it has not been possible to correlate clinical phenotypes with *TET2* mutations (for example, regarding their precise location or heterozygosity). Other questions relate to the dependency of malignant cells on the initial *TET2* mutation, and why some patients with *TET2* mutations remain healthy while others develop a myeloid or a lymphoid malignancy [123-126]. Addressing such questions is important, not only with regard to mutations in genes involved in the control of DNA methylation (such as TET2 or DNMT3A), but also for mutation in genes controlling other functions that predate and may predispose to the development of adult malignancies [125-127].

## Abbreviations

2HG: 2-hydroxyglutarate
5-caC: 5-carboxycytosine
5-fC: 5-formylcytosine
5-hmC: 5-hydroxymethylcytosine
5-hmU: 5-hydroxymethyluracil
5-mC: 5-methylcytosine
αKG: α-ketoglutarate
AID: Activation-induced deaminase
AITL: Angioimmunoblastic T-cell lymphoma
AML: Acute myeloid leukemia
BER: Base-excision repair
COSMIC: Catalogue of somatic mutations in cancer
DNMT: DNA methyltransferase
ES: Embryonic stem
FH: Fumarate hydratase
IDH: Isocitrate dehydrogenase
MBD: Methyl-binding domain
MBP: Methyl-CpG binding
MDS: Myelodysplastic syndrome
miRNA: microRNA
MPN: Myeloproliferative neoplasms
OGT: O-linked β-D-N acetylglucosamine transferase
PRC2: Polycomb repressive complex 2
SDH: Succinate dehydrogenase
TDG: Thymidine DNA glycosylase
TET: Ten-eleven translocation
UTR: Untranslated region
